# Peripartum cardiomyopathy: disease or syndrome?

**DOI:** 10.1136/heartjnl-2018-314252

**Published:** 2019-02-12

**Authors:** Lucia Baris, Jérôme Cornette, Mark R Johnson, Karen Sliwa, Jolien W Roos-Hesselink

**Affiliations:** 1 Department of Cardiology, Erasmus MC, Rotterdam, The Netherlands; 2 Department of Obstetrics and Gynaecology, Erasmus MC, Rotterdam, The Netherlands; 3 Department of Gynaecology and Obstetric Medicine, Imperial College London, London, UK; 4 Department of Cardiology, Hatter Institute for Cardiovascular Research in Africa, Cape Town, South Africa

**Keywords:** pregnancy, heart failure, familial cardiomyopathies, idiopathic dilated cardiomyopathy

## Abstract

Peripartum cardiomyopathy (PPCM) is a rare form of pregnancy-associated heart failure and is considered to be a diagnosis of exclusion. There are many hypotheses on the aetiology of PPCM; however, the exact pathophysiological mechanism remains unknown. It shows many resemblances to other conditions, such as familial dilated cardiomyopathy or myocarditis, and therefore it can be hard to make a definite diagnosis. We describe four cases of peripartum-onset heart failure in women who were suspected of having PPCM. We discuss the differential diagnosis, pathophysiological mechanisms and various diagnostic modalities.

## Introduction

Peripartum cardiomyopathy (PPCM) is a rare form of pregnancy-associated heart failure and is considered to be a diagnosis of exclusion. Currently, the Heart Failure Association of the European Society of Cardiology Working Group on PPCM defines it as an idiopathic cardiomyopathy presenting with heart failure secondary to left ventricular systolic dysfunction towards the end of pregnancy or in the months following delivery, where no other cause of heart failure is found.^1^ The left ventricle may not be dilated, but the ejection fraction has to be reduced below 45%.^2^

PPCM may show resemblances to dilated cardiomyopathy (DCM), in clinical presentation as well as in genetic background, and it is associated with maternal and fetal mortality and morbidity.^2^ Several pathophysiological mechanisms have been proposed to be involved in the aetiology of PPCM, such as autoimmunity, (viral) myocarditis, maladaptation to the hyperdynamic state of pregnancy, prolonged tocolysis and prolactin mediation.^3–5^ Also, the high variation in incidences between countries and continents suggests environmental factors to play a role.^4^

Patients are treated with conventional heart failure medication. Recently, bromocriptine has been discovered as having positive effects on the outcomes and can be added to standard heart failure treatment. While evidence on the effectivity and safety of bromocriptine is scarce, its use has been associated with a decrease in morbidity and mortality rates in patients with PPCM and higher rates of left ventricular recovery.^6^ Although full recovery does occur, a significant proportion of patients suffer from persistent systolic ventricular dysfunction.

The diagnosis of PPCM is difficult to make, as it is a diagnosis of exclusion, with a considerable overlap with other conditions. Especially in women who present with acute heart failure at the end of pregnancy or directly post partum, thorough investigations and intensive follow-up may often lead to alternative diagnoses. We describe four cases of patients with peripartum heart failure at our centre who were initially diagnosed with PPCM, but in whom a different diagnosis eventually emerged. We discuss the differential diagnosis, pathophysiological mechanisms and the use of various diagnostic modalities.

### Case A

**Figure 1:**
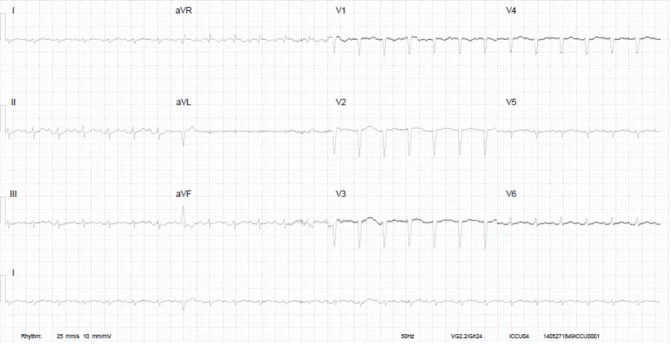
ECG of patient A at admission. There was a noted sinus tachycardia with low voltages in the extremity leads and a QS pattern in V1–V3. aVR, augmented vector right; aVL, augmented vector left; aVF, augmented vector foot.

This 31-year-old primigravida woman with a history of pulmonary embolism and multiple deep venous thrombosis during the index pregnancy was admitted to the obstetric department with pre-eclampsia with severe hypertension of 160/100 mm Hg at 26 weeks of pregnancy. She had been taking vitamin K antagonists, which were switched to low-molecular-weight heparin, and she was treated with magnesium sulfate and intravenous antihypertensive drugs. A primary caesarean section under general anaesthesia was performed at 30 weeks of pregnancy due to fetal distress, delivering a growth-restricted, premature girl of 920 g. Postsurgery the mother developed severe postpartum haemorrhage with a total of 5000 mL blood loss, and treatment was complicated by her anticoagulation therapy. She received blood transfusions and was discharged from the hospital with a haemoglobin level of 77.3 g/L. Three days later she was readmitted with complaints of fatigue, severe dyspnoea and fever. She had pleural effusion and thrombocytopaenia. She was later transferred to the cardiac intensive care unit (ICU) due to deterioration of her condition, and echocardiographic examination revealed severe left ventricular dysfunction with mild dilatation of the ventricles. ECG showed a sinus tachycardia with low voltages in the extremity leads and a QS pattern in V1–V3 (figure 1). Coronary angiography showed normal coronary arteries and she was diagnosed with PPCM. She soon went into cardiogenic shock with high lactate levels, and when high dosages of inotropes could not stabilise her extracorporeal membrane oxygenation (ECMO) was started. Heart failure medication was started, which stabilised her condition, and after 15 days the ECMO could be removed. ECMO was complicated by cannula-related severe ischaemia of her right foot, which had to be amputated. She was transferred to a rehabilitation clinic after hospital discharge.

Because of the combination of multiple venous emboli, pre-eclampsia with proteinuria, pleural effusion and the thrombocytopaenia, the patient was referred to an immunologist, who diagnosed her with antiphospholipid syndrome and systemic lupus erythematosus (SLE). Her last echocardiogram showed only a mild impairment of left ventricular function.

### Case B

**Figure 2:**
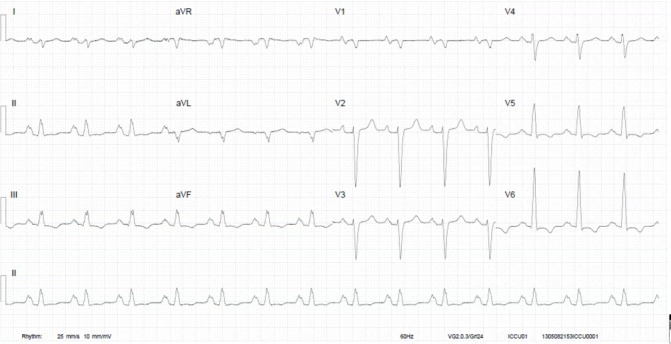
ECG of patient B at diagnosis. Sinus rhythm with notched high-amplitude P-waves, QRS of 120 ms, negative repolarisation in the inferior and lateral leads, and QS complex in V1. aVR, augmented vector right; aVL, augmented vector left; aVF, augmented vector foot.

**Figure 3:**
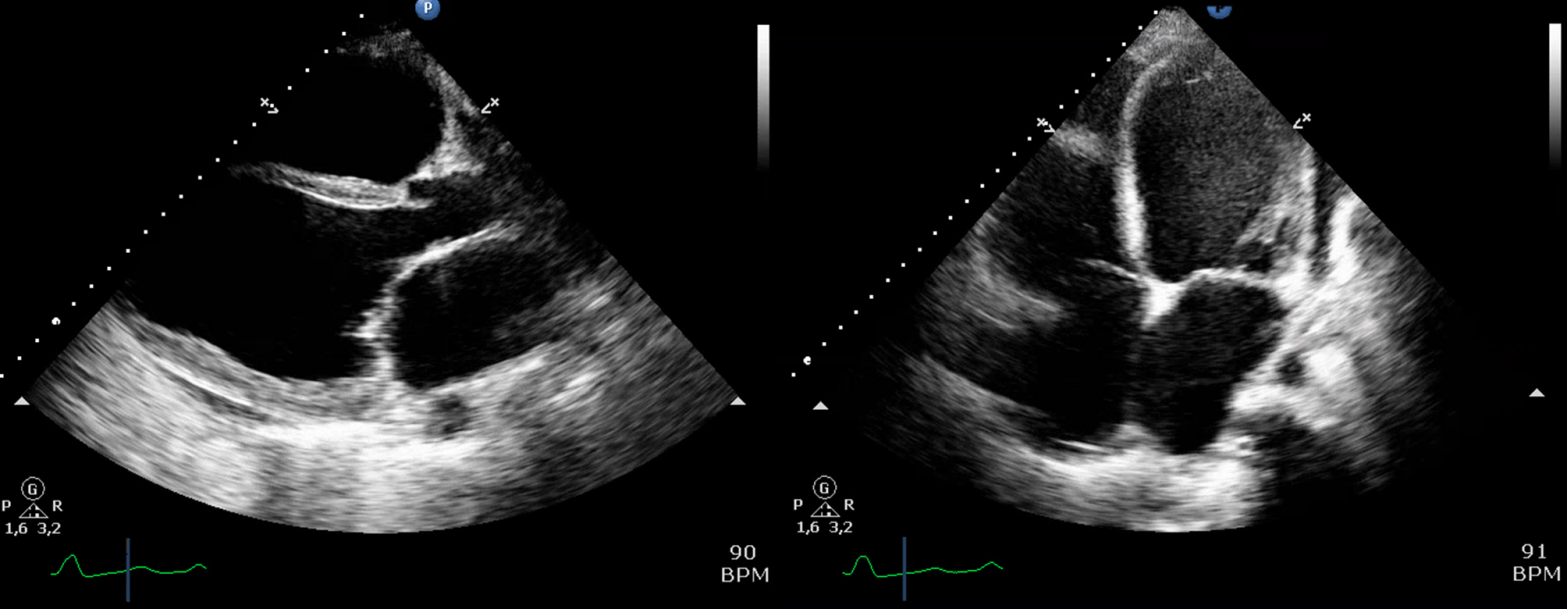
(A) Parasternal long axis on echocardiogram of patient B at diagnosis. The LVEDD was 76 mm. (B) Four-chamber apical view of patient B at diagnosis. Biventricular dilatation with severely impaired right and left ventricular function. Severe mitral regurgitation. BPM, beats per minute; LVEDD, left ventricular end diastolic diameter.

This 25-year-old primigravida woman with a history of childhood asthma and generalised anxiety disorder was admitted to a cardiology ward unit with progressive dyspnoea at 26 weeks of pregnancy. Her ECG revealed several abnormalities (figure 2), and echocardiography showed a DCM with severe left and right ventricular dilatation and a left ventricular ejection fraction of about 10%–15% (figure 3A,B). She underwent emergency caesarean section the following day and was admitted to the ICU afterwards, where she was treated with heart failure medication and inotropes. Low-molecular-weight heparins were started because of a thrombus in the right ventricular outflow tract. ECMO was started due to refractory cardiogenic shock, and later a left ventricular assist device was implanted. There was no evidence of coronary artery disease. Due to severe confusion and drowsiness in the ICU, a CT of the brain was made, showing generalised brain atrophy. Therefore, a metabolic origin was suspected. Myocardial biopsy showed a myopathic image suspected for mitochondrial storage disease due to excessive glycogen storage. Morbus Pompe and Fabry were ruled out and viral tests were negative, as were autoimmune and paraneoplastic syndromes. Quadriceps femoris muscle biopsy showed a myopathy without specific characteristics. Blood and urine tests were performed, as well as genetic tests. Plasma carnitine was strongly elevated and a diagnosis of succinyl-CoA ligase deficiency was suspected, but could eventually not be confirmed as the results of further tests and analyses were inconclusive. No known mutations in the DCM genes were found, and the diagnosis of PPCM remained the working diagnosis. Her prematurely born son was diagnosed with (congenital) hypertrophic cardiomyopathy. One year later, the patient underwent a cardiac transplantation and is currently doing relatively well.

### Case C

**Figure 4:**
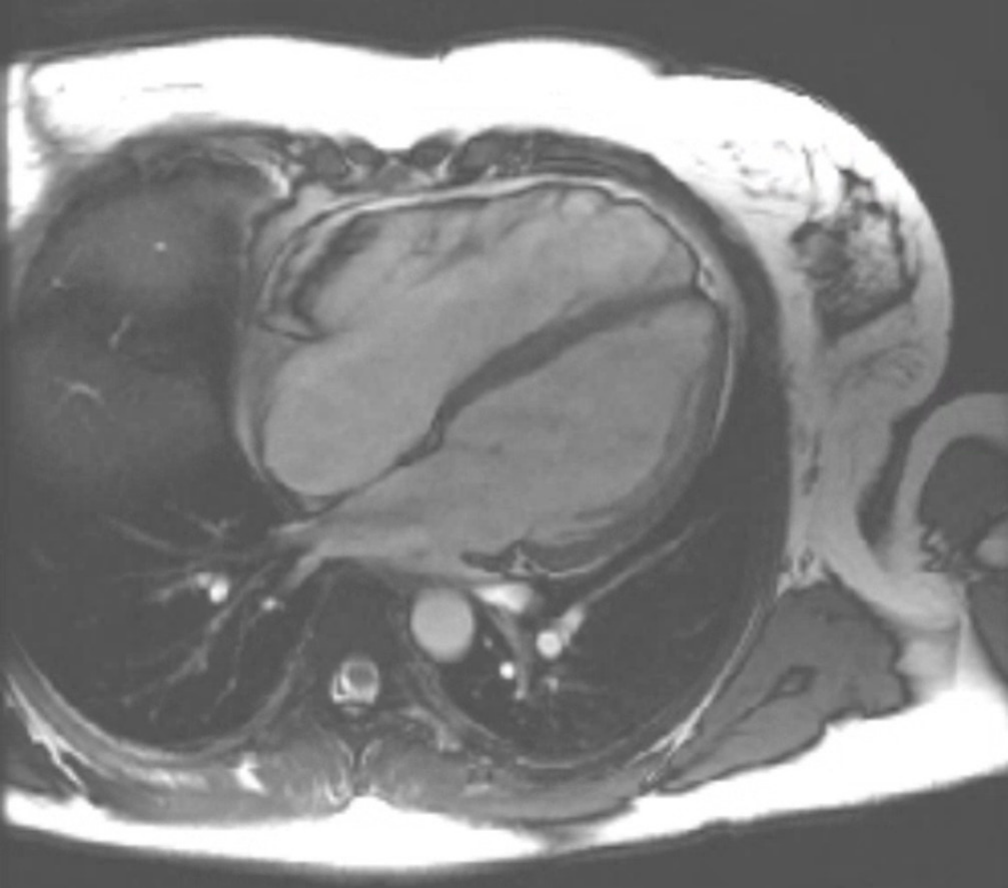
Four-chamber MRI view of patient C. Distinct tubular dilatation of the right ventricle (RV) with dyskinesia, impaired RV ejection fraction (34%) and elevated RV end-diastolic volume index (132.4 mL/min). Also oedema and late enhancement in the wall of the right ventricle.

This 36-year-old woman with a history of unspecified palpitations and a family history of sudden cardiac death, who had once been assessed by a cardiologist because of suspected long-QT syndrome in her infant daughter, was admitted to the cardiology ward unit with complaints of dyspnoea and chest pain, 4 months after giving birth to her third child. Her N-terminal prohormone of brain natriuretic peptide (NT-proBNP) was markedly elevated, and echocardiography revealed a DCM with a left ventricular ejection fraction of 15% and moderate to severe tricuspid insufficiency and a moderate to severe mitral insufficiency. She was diagnosed with PPCM and was treated with heart failure medication on which she slowly recovered. She was discharged home and seen by a cardiologist for regular follow-up. A 24-hour Holter ECG showed frequent, non-sustained ventricular tachyarrhythmias, and as part of her outpatient follow-up an MRI scan of her heart was made, which showed distinct tubular dilatation of the right ventricle, with less distinct dilatation of the left ventricle (figure 4). Hypokinesia was most prominent in the right ventricle and there was severe tricuspid insufficiency. A diagnosis of arrhythmogenic right ventricular cardiomyopathy (ARVC) was made and a prophylactic internal cardiac defibrillator was implanted. Genetic tests revealed no known mutation. Her left ventricular function has almost fully recovered, but her right ventricular function remains moderately impaired.

### Case D

This 26-year-old primigravida woman in labour with an unremarkable medical history was urgently admitted to the obstetrics department with failure to progress in the second stage and the suspicion of an intrauterine infection. She underwent an emergency caesarean section, which was complicated by a severe postpartum haemorrhage with a blood loss of 2 L. She was treated with sulprostone and intravenous fluids and a blood transfusion, immediately after which she became severely dyspnoeic. Chest X-rays showed congestion, and echocardiography revealed severe left ventricular dysfunction with an estimated ejection fraction of 15% and only moderate wall motility in the basal septal region. There were no signs of ischaemia or coagulopathy (thereby excluding amniotic fluid embolism). The diagnosis of PPCM was suspected and she was intubated and transferred to the ICU, where she was treated with conventional heart failure medication. She could be extubated very quickly after initiation of therapy, and repeat echocardiography showed normalisation of left ventricular function with normal ventricular dimensions within a few days. She had experienced the caesarean section as very traumatic and has visited a psychologist to help her cope. In the outpatient clinic, the question rose whether she had suffered from PPCM or whether her acute heart failure was caused by a takotsubo cardiomyopathy. The urgent and complicated characteristics of the delivery, as well as the echocardiographic images of akinesia affecting the whole ventricle except the basal segments and the very rapid recovery of the ejection fraction, are suggestive of a takotsubo cardiomyopathy rather than a PPCM. She returned to the cardiologist a few years later with the wish to become pregnant again. Due to her recovered left ventricular function, there were no objections to a subsequent pregnancy and she has had several uncomplicated pregnancies since. Her left ventricular function has remained normal to date.

## Discussion

We have described four women who presented during pregnancy or in the early postpartum period with signs of heart failure and echocardiographic signs of severely impaired left ventricular function. The peripartum onset makes the diagnosis of PPCM likely; however, features of the patients’ history and the results of specific investigations including histology suggested alternative diagnoses.

Myocarditis in SLE is usually asymptomatic, and while cardiac problems in SLE are often related to coronary disease, which was ruled out in our patient, lupus myocarditis without coronary involvement is not uncommon.^7^ Moreover, the incidence of non-coronary cardiac disease in patients with SLE is relatively common, certainly when compared with the incidence of PPCM in the general population, and this is an important consideration in making a diagnosis in such a complex patient.^7^ Also, lupus flares as a result of pregnancy are not uncommon. The distinction between PPCM and SLE-related or other myocarditis is challenging, but very important with respect to subsequent pregnancies.^8^ In contrast to lupus myocarditis, PPCM has a high recurrence rate and persisting ventricular dysfunction is common, both make the management of future pregnancies challenging.^8^

Mitochondrial myopathies can be isolated to skeletal muscle, but can also affect cardiac muscle, presenting with a wide range of clinical problems.^9^ As with other metabolic disorders, mitochondrial myopathies can present during periods of increased physiological stress, such as pregnancy and delivery,^9^ with acute heart failure or encephalopathy. In our patient, the extensive cerebral atrophy was remarkable, as well as the marked cardiac hypertrophy in her newborn son. While she still remains without definitive diagnosis, a mitochondrial problem would be consistent with the clinical picture.

Other forms of cardiomyopathy, such as ARVC as diagnosed in our patient, have been misdiagnosed as PPCM when presenting in the peripartum period. In an article from 2011, mutations in the titin (TTN) gene were associated with ARVC, the same mutation as reported in both DCM and PPCM.^10 11^ There are numerous case reports describing the first presentation of these cardiomyopathies during the peripartum period, consistent with a common aetiological pathway.

In PPCM, many pathophysiological mechanisms have been suggested, but it is unlikely that there is one single mechanism. With respect to the pregnancy-associated aetiologies, many advances have been made in the angiogenic imbalance theory.^12 13^ During pregnancy, the placenta secretes antiangiogenic factors, such as soluble fms-like tyrosine kinase-1 (sFLt-1). This factor is important in the regulation of blood vessel formation in various tissues, and has been linked to (pre-)eclampsia and heart failure.^14 15^ sFLt-1 causes ventricular dysfunction, the extent of which correlates with the serum quantity of sFLt-1, and it is known that women with PPCM have extremely high circulating levels.^13^ The profound systolic impairment found in women with PPCM is mimicked in mice lacking PGC-1α (peroxisome proliferator-activated receptor gamma coactivator 1-alpha, a powerful regular of angiogenesis) after administration of exogenous sFLt-1, suggesting an aetiological role for excess antiangiogenic factors in the peripartum period.^16^

Another hypothesis that has been repeatedly proposed within the recent literature is the prolactin and oxidative stress theory.^1 4^ Late in pregnancy, high levels of low-density lipoprotein-cholesterol are present, which are susceptible to oxidation. Oxidative stress in pregnancy in combination with high levels of prolactin at the end of pregnancy can result in the formation of the 16 kDA form of prolactin, which is both angiostatic and proapoptotic, and can impair endothelial function resulting in cardiac inflammation.^2 4 17^ High prolactin levels are inevitable in pregnancy, thus a pathophysiological role in PPCM is very possible; however, it might not be exclusive to pregnancy. Hyperprolactaemia is present in about 25% of non-pregnant patients with heart failure and also seems to be a predictor of prognosis in advanced congestive heart failure in non-pregnant patients.^17–20^

The introduction of bromocriptine as treatment for PPCM is based on the theory of prolactin-mediated endothelial dysfunction; it seems to have beneficial effects on morbidity and mortality and is associated with higher rates of left ventricular recovery.^6^ However, in women taking bromocriptine for the prevention of breast engorgement in the postpartum period, there was an increased incidence of myocardial infarction and other thromboembolic events.^21 22^ Therefore, concomitant anticoagulation therapy is strongly recommended.^4 22^ Outside of pregnancy, bromocriptine also reduces cardiovascular mortality in other conditions.^23^ Bromocriptine has been shown to lower plasma norepinephrine levels and to induce vasodilation acting via dopaminergic receptors in the vessel wall, suggesting alternative mechanisms for its beneficial effects independent of any effect on prolactin levels.

**Figure 5:**
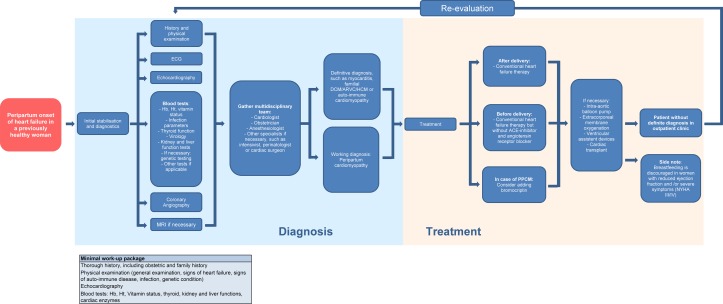
Flow chart for management of peripartum onset heart failure. ARVC, arrhythmogenic right ventricular cardiomyopathy; DCM, dilated cardiomyopathy; PPCM, peripartum cardiomyopathy; HCM, hypertrophic cardiomyopathy; Hb, hemoglobin; Ht, hematocrit; NYHA, New York Heart Association.

Due to PPCM being a diagnosis of exclusion and the overlap with other syndromes, it is possible that many cases of PPCM are missed. On the other hand, the haemodynamic stress of pregnancy can reveal undiagnosed cardiac conditions such as DCM, or can give rise to conditions such as takotsubo cardiomyopathy, which could also mimic PPCM. It can also be hard to distinguish PPCM from pre-eclampsia and eclampsia, especially when they are complicated by pulmonary oedema. Furthermore, drugs administered for postpartum haemorrhage, like prostaglandin analogues, can cause coronary spasms and other severe cardiovascular reactions, mimicking PPCM.^24^ Consequently, many women diagnosed with PPCM, when they present with (acute) heart failure during pregnancy or in the postpartum period, may have their diagnosis changed after additional tests. Thus, the importance of re-evaluation of a diagnosis of PPCM is clear. Figure 5 depicts a flow chart for the management of previously healthy women with a peripartum onset of heart failure, in which re-evaluation of the working diagnosis is incorporated, including a minimal work-up package for diagnostics.

Around two-thirds of women with PPCM present post partum,^25^ a finding that can therefore also be suggestive of the diagnosis: in the case of a previously discharged healthy women presenting with heart failure a few months after delivery, the diagnosis of PPCM becomes more probable. However, the patients we describe in this paper highlight that, in some cases, especially when there are atypical symptoms or an onset before 36 weeks of gestation, an alternative diagnosis is more likely. Table 1 gives an overview of various atypical presentations of peripartum-onset acute heart failure with corresponding differential diagnoses. Overall in the literature, about 15% of patients with PPCM have a genetic mutation known to be associated with DCM. This may seem to be lower than expected, but a genetic mutation is found in only 30% of DCM cases. Although data are scarce, there are an increasing number of reports available in the literature in which a connection between PPCM and familial DCM is suggested. In one Dutch study, 90 families with familial DCM were studied, looking for evidence of PPCM; in addition, patients with PPCM and their first-degree relatives were genetically screened. The authors concluded that at least a proportion of PPCM cases are the initial presentation of familial DCM.^26^

**Table 1:** Differential diagnosis of peripartum cardiomyopathy-atypical presentations of acute heart failure in the peripartum period

Atypical presentation of peripartum-onset heart failure	Differential diagnosis
Presentation before 36th week of pregnancy	Pre-existing (dilated) cardiomyopathy, pulmonary oedema secondary to (pre-)eclampsia, myocarditis, autoimmune disease-related myocardial dysfunction (systemic lupus erythematosus, antiphospholipid syndrome), metabolic myopathy, ischaemic heart disease (eg. spontaneous coronary artery dissection).
Presentation after 6 months postdelivery	Familial, pre-existing (dilated) cardiomyopathy, myocarditis.
Presentation during complicated or traumatic delivery	Amniotic fluid embolism, takotsubo cardiomyopathy.
Acute presentation immediately post partum after misoprostol administration	Coronary artery spasm with subsequent cardiac dysfunction, amniotic fluid embolism.
Primary presentation with (tachy)arrhythmias or (aborted) sudden cardiac death	Arrhythmogenic right ventricular cardiomyopathy, catecholaminergic polymorphic ventricular tachycardia, mitral valve prolapse.

This identification of pathogenic mutations in other familial cardiomyopathies further highlights the possibility that many cases of PPCM might be due to genetic DCM revealed for the first time by the stress of pregnancy. Also, the higher incidence in specific ethnic groups and in defined areas of the world suggests a role of environmental and/or genetic factors.^4^ Furthermore, the diagnosis of takotsubo cardiomyopathy has to be considered in the event of a traumatic or complicated delivery, as well as in the critically ill (severe pre-eclampsia or eclampsia, severe postpartum haemorrhage) or postoperative patient (caesarean delivery).^27^

Due to the difficulties in diagnosing heart failure manifesting during pregnancy or in the postpartum period and the limited availability of broad genetic tests, it is very likely that patients with genetic forms of DCM and other cardiomyopathies have been included in the published studies on PPCM over the past decades. This has the potential to fuel a cycle of overdiagnosis and ever increasing incidence of what might be a relatively rare condition. For example, the increases in incidence of PPCM in the USA (from 1 per 4350 in 1990–1993 to 1 per 2229 in 2000–2002)^28^ might be attributable to increased awareness of the condition, but also to (subsequent) overdiagnosis.

It is probable that a collection of pregnancy-related factors, which include haemodynamic stress and high prolactin levels, combined to varying degrees with an underlying genetic predisposition for cardiomyopathy, result in the prepartum or immediate postdelivery onset of heart failure, with the exact importance of each factor varying from case to case. As the exact pathophysiology remains largely unknown and as it displays very similar clinical, demographical and genetic characteristics to other forms of cardiomyopathy, it may be defensible to regard PPCM as a syndrome, rather than a distinct diagnosis—a syndrome of peripartum heart failure of unknown origin, characterised by a reduced left ventricular ejection fraction accompanied by symptoms of heart failure, elicited by peripartum-induced changes in haemodynamic, hormonal and/or homeostatic systems, in previously healthy women, women with a pre-existent (unknown) condition or women with a predisposition for developing (dilated) cardiomyopathy. As such, it remains a diagnosis of exclusion or a diagnosis made in the early stages of the disease, when more definite answers are still being sought.
